# Design and Synthesis of Fe_3_O_4_-Loaded Polymer Microspheres with Controlled Morphology

**DOI:** 10.3390/polym17070865

**Published:** 2025-03-24

**Authors:** Talya Scheff, Florence Acha, Nathalia Diaz Armas, Sevil Turkoglu, Jinde Zhang

**Affiliations:** Department of Plastics Engineering, University of Massachusetts Lowell, Lowell, MA 01854, USA; talya_scheff@student.uml.edu (T.S.); florence_acha@student.uml.edu (F.A.); nathalia_diazarmas@student.uml.edu (N.D.A.); sevil_kaynar@student.uml.edu (S.T.)

**Keywords:** polymer composites, magnetic materials, mini-emulsion, γ-ray irradiation

## Abstract

A novel approach for synthesizing polymer particles that encapsulate Fe_3_O_4_ nanoparticles using γ-ray irradiated mini-emulsion polymerization is presented. To achieve high encapsulation efficiency, the surface properties of Fe_3_O_4_ nanoparticles are modified from hydrophilic to hydrophobic, allowing for effective dispersion in the monomer prior to polymerization. Mini-emulsion techniques have proven essential for forming the encapsulated structure and controlling particle size distribution. γ-ray irradiation initiated radical polymerization and solidified the emulsion structure. Characterization via FTIR, XRD, and TEM confirmed the successful encapsulation of Fe_3_O_4_ nanoparticles within polymer particles. Magnetic measurements demonstrated superparamagnetic behavior and high magnetic induction, both of which are properties considered advantageous for biomedical applications.

## 1. Introduction

Magnetic polymer composite particles (MPCPs) have attracted considerable interest due to their multifunctional characteristics, which integrate magnetic properties with polymeric microspheres or particles. Their versatile characteristics hold significant potential for applications in various fields, such as biomedical engineering, bioengineering, hyperthermia therapy, magnetic resonance imaging (MRI), and enzyme immobilization [1,2,3,4,5,6,7,8,9]. The synthesis of MPCPs is typically achieved through three main approaches: physical embedding, in situ synthesis, and monomer polymerization.

In the physical embedding method, magnetic nanoparticles are dispersed within a polymer solution or melt and then undergo processes such as atomization, deposition, or solvent evaporation to form composite particles. Although this technique is relatively simple, achieving uniform particle size and shape remains a challenge, which is critical for many applications that require uniform magnetic responses. Furthermore, this method is limited to soluble or meltable polymers and can be energy-intensive. In situ synthesis, pioneered by Ugelstad [10], can produce MPCPs with a narrow size distribution. However, the synthesis process is much more complicated. Recently, monomer polymerization has become a prevalent method for preparing MPCPs that encapsulate magnetic nanoparticles inside polymers due to its versatility, cost-effectiveness, and ability to control particle size [11,12,13,14,15,16,17,18,19]. Various strategies, including conventional emulsion polymerization, inverse emulsion/microemulsion polymerization, soapless emulsion polymerization, mini-emulsion polymerization, precipitation polymerization, ATRP, and other techniques, have been employed. However, for all of the above strategies, the achieved encapsulation rate is not desirable, leading to final products that contain not only MPCPs encapsulating magnetic nanoparticles but also others, such as pure polymer particles (PPPs) and exposed or bare magnetic particles (BMPs). For biomedical applications, MPCPs must satisfy several essential criteria, including superparamagnetic behavior, consistent and uniform particle size, stability without sedimentation, high and reproducible magnetic content, non-toxicity, and minimal iron leakage [20]. The formation of PPPs and BMPs is undesirable, as it can reduce magnetic performance, increase iron leakage, and lead to a broader particle size distribution. Thus, a major challenge lies in preventing the formation of PPPs and BMPs while ensuring the magnetic nanoparticles are well encapsulated with a uniform and narrow size distribution.

In this paper, mini-emulsion polymerization was performed to prepare MPCPs encapsulating Fe_3_O_4_ nanoparticles. Divinylbenzene (DVB) was chosen as a monomer due to its high crosslinking ability, rapid polymerization, and effective encapsulation. Its two vinyl groups enable a rigid poly(divinylbenzene) (PDVB) shell, preventing Fe_3_O_4_ agglomeration and ensuring uniform coating. The high crosslinking density suppresses phase separation, enhancing structural integrity. Additionally, PDVB improves chemical and thermal stability, making the composite more durable. These properties make DVB ideal for fabricating stable Fe_3_O_4_/polymer hybrid materials with strong encapsulation and dispersion stability. The radical polymerization of DVB was initiated via γ-radiation at room temperature, and the polymerization degree was controlled by easily changing irradiation time. Moreover, products synthesized using γ-radiation are well suited for biomedical applications, as they remain free from contamination by chemical initiators and are inherently sterilized. At last, the mechanism for forming MPCPs with encapsulated Fe_3_O_4_ nanoparticles was analyzed to identify optimal synthesis conditions.

## 2. Experimental

### 2.1. Materials

Divinyl benzene (DVB) was purified by passing it through an alumina column to remove the radical inhibitor. After purification, the DVB was stored at approximately 4 °C until use. The other materials used in this study, including ferrous sulfate heptahydrate (FeSO_4_·7H_2_O), ferric chloride (FeCl_3_), 25 wt.% aqueous ammonia, oleic acid, acetyl alcohol (CA), sodium dodecyl sulfate (SDS), and ethanol, were of analytical grade and were used without further purification.

### 2.2. Magnetic Nanoparticle (MNP) Synthesis and Modification

Magnetic nanoparticles (MNPs) were synthesized based on a modified version of a previously reported method [21]. Three types of MNPs were prepared by adjusting the reaction parameters, as summarized in Table 1. In a typical procedure, 7.31 g of ferric chloride (FeCl_3_) and 8.35 g of ferrous sulfate heptahydrate (FeSO_4_·7H_2_O) were dissolved in 30 mL of distilled water to form a uniform solution. This mixture was then diluted with an additional 70 mL of distilled water and heated to 85 °C under constant stirring. Precipitation was initiated by adding 30 mL of 25% (*w*/*w*) aqueous ammonia (NH_3_·H_2_O) to the heated solution. After one minute, 2 g of oleic acid was dropped into the solution to modify the particle surface. This modification reaction was maintained for 3 h, after which the black magnetic particles were collected using a magnet. Then, the collected particles were washed three times with distilled water and ethanol via magnetic decantation to remove any residual impurities. The purified MNPs were dried in a vacuum oven at 120 °C for 24 h. Finally, the dried MNPs were stored in sealed bottles at room temperature for future use.

### 2.3. Preparation of MPCPs

In a typical synthesis, 0.10 g of magnetic nanoparticles (MNPs) and 0.2 g of acetyl alcohol (CA) were mixed with 3 g of divinyl benzene (DVB) monomer. The mixture was dispersed by ultrasonication to achieve a uniform distribution of the components. Separately, 0.06 g of SDS was dissolved in 30 mL of distilled water. Once both mixtures were homogeneous, the oil phase was added to the aqueous phase under vigorous stirring at 1000 rpm for 1 h, forming a primary emulsion. This emulsion was then refined through mini-emulsification by ultrasonication in a water bath for 10 min, with ice added to maintain a low temperature and prevent premature polymerization. Before initiating polymerization, nitrogen gas was bubbled through the mini-emulsion to eliminate oxygen, and the mixture was sealed in a 100 mL narrow-neck glass bottle. Polymerization was triggered using γ-ray irradiation from a ^60^Co source at 23 °C, with a dose rate of 60 Gy/min and a total absorbed dose of 86.4 kGy. After polymerization, the magnetic products were separated using a magnetic field for 20 min. The supernatant was carefully decanted and preserved for reference characterization. The MPCPs were then repeatedly washed with distilled water and ethanol using magnetic decantation to remove any remaining reactants and byproducts. Finally, the purified microspheres were air-dried at room temperature.

To study the effect of SDS and CA on the final morphology of MPCPs, a series of experiments were carried out, and the specific conditions were listed in Table 1.

### 2.4. Characterizations

In this work, Fourier transform infrared (FTIR) spectroscopy with samples prepared using the KBr pellet method was used to characterize the surface modification of MNPs and the chemistry composition of prepared MPCPs. Water Contact Angle (WCA) measurement via a Contact Angle Meter SL200B (Solon Tech. Co., Ltd., London, UK) was used to indicate the effectiveness of the oleic oil modification on MNPs. The crystal structure and phase composition of the MNPs were examined by powder X-ray diffraction (XRD) using a Japan Rigaku D/max γ_A_ diffractometer, scanning at a rate of 0.02 degrees per second over a 2θ range of 20° to 80°. Thermogravimetric analysis (TGA) was conducted with a Shimadzu TGA-50H instrument to evaluate the oleic acid content on the magnetic nanoparticles and determine the MNP content in MPCPs. For TGA, the samples were heated from 50 °C to 700 °C at a rate of 10 °C per minute in air, with weight loss monitored as a function of temperature. To examine the morphology of the MPCPs, transmission electron microscopy (TEM) was employed using a Hitachi model H-7650 with an accelerating voltage of 200 kV. The magnetic properties of both MNPs and MPCPs were measured using an MPMS XL magnetometer at room temperature. The applied magnetic field varied between 0, 1, 0, −1, and 0 T during the measurement process.

## 3. Results and Discussion

### 3.1. Characterization of Synthesized MNPs

**Scheme 1:**
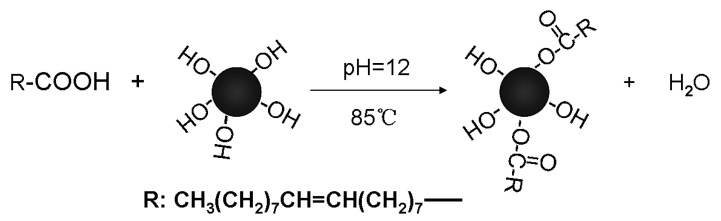
Illustration of oleic acid modification on MNPs.

**Figure 1:**
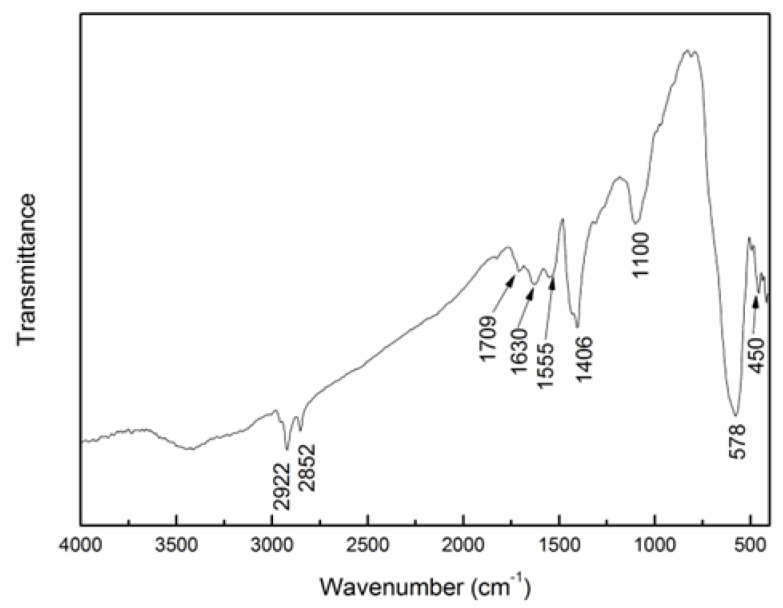
FTIR spectrum of oleic acid-modified MNPs.

The surface modification of the synthesized MNPs with oleic acid was confirmed by the FTIR spectrum of the MNPs (see Figure 1), and the corresponding reaction is shown in Scheme 1. The peaks at 578 and 450 cm^−1^ correspond to the vibrations of Fe^2^⁺–O^2−^ and Fe^3^⁺–O^2−^, respectively. The peaks at 2922 and 2850 cm^−1^ are attributed to the stretching vibrations of saturated –C–H bonds in the oleic ester. The band at 1709 cm^−1^ is associated with the stretching vibration of the C=O group (νC=O) in the oleic ester, while the peak at 1110 cm^−1^ reflects the characteristic stretching vibration of the C–O–C group in the ester. The bands at 1630, 1555, and 1406 cm^−1^ likely result from the formation of oleate adsorbed on the MNPs’ surface.

**Figure 2:**
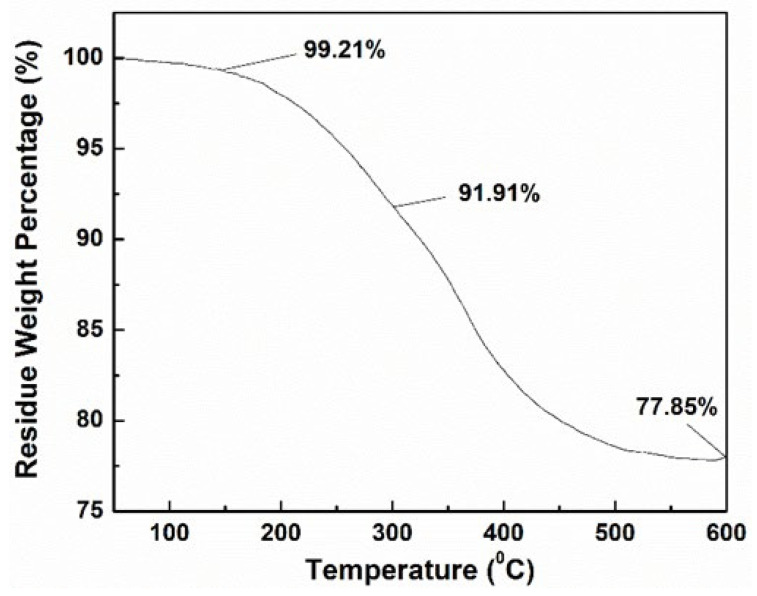
TGA curve of oleic acid-modified MNPs.

TGA was conducted to determine the oleic acid content on the MNPs, and the result is shown in Figure 2. The analysis showed multiple stages of weight loss. Initially, up to 150 °C, a 0.79% weight loss was observed, likely due to the evaporation of moisture. Between 150 °C and 300 °C, a 7.3% weight loss occurred, attributed to the removal of physically absorbed oleic acid. From 300 °C to 600 °C, an additional 14.06% weight loss was observed, corresponding to the decomposition of oleic ester. The final residual weight was 77.85%, indicating the amount of pure magnetic nanoparticles (MNPs). Based on these findings, the total amount of oleic acid modified onto the MNPs was calculated to be 21.53%.

**Figure 3:**
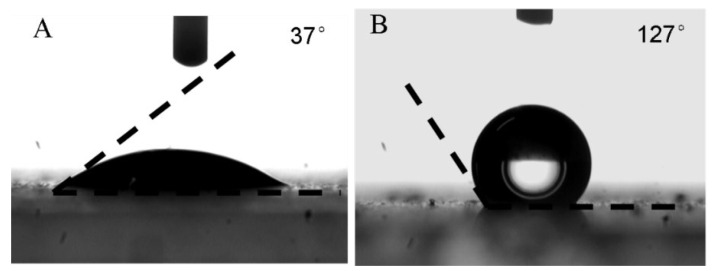
WCA on MNPs (**A**) unmodified with oleic acid; (**B**) modified with oleic acid.

**Figure 4:**
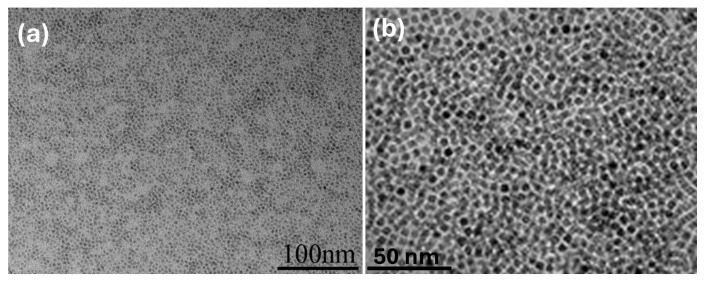
TEM image of (**a**) oleic acid-modified MPs in divinylbenzene; (**b**) oleic acid-modified MPs without DVB.

In theory, increasing the amount of oleic acid on the MNP surface should result in enhanced hydrophobicity. In this work, the surface-wetting properties of the oleic acid-modified MNPs were quantitatively evaluated by measuring the water contact angle (WCA) on the MNP surface. To prepare for the measurement, an MNP suspension was spin-coated onto a glass slide and air-dried to create a smooth, flat surface. The results of these measurements are shown in Figure 3. As shown, the unmodified MNPs exhibited a hydrophilic surface with a WCA of 37°. After modification with oleic acid, the WCA increased significantly to 127°, indicating a successful shift from a hydrophilic to a hydrophobic surface. This enhanced hydrophobicity was essential in determining the final encapsulated morphology of MPCPs. As seen in Figure 4a, the MNPs dispersed well in the DVB monomer, further confirming the increased hydrophobicity of the modified MNPs, which allowed for easier dispersion in the oil phase. The average size of dispersed MNPs shown in Figure 4a,b is about 5 ± 1 nm.

### 3.2. Morphology Characterization of MPCPs

**Figure 5:**
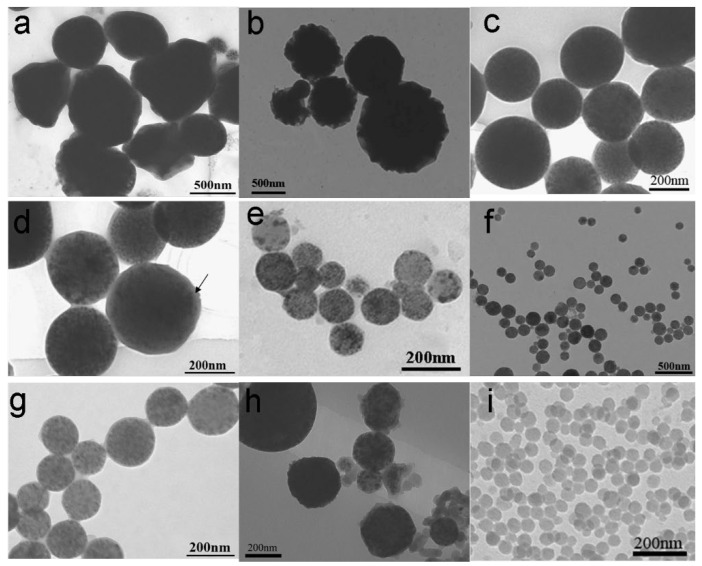
TEM images of (**a**) S-1, (**b**) S-2, (**c**) S-3, and (**d**) S-3 at higher magnification; (**e**) S-4, (**f**) S-5, and (**g**) S-5 at higher magnification; and (**h**) S-6 and (**i**) S-7, as listed in Table 1.

To optimize MPCP parameters and elucidate the factors governing their morphology, additional experiments were conducted with detailed formulations listed in Table 1. The results presented in Figure 5 reveal three key factors influencing the morphology of the MPCPs: the hydrophobicity of the MNPs, the content of SDS, and the amount of CA. Highly hydrophilic MNPs, lacking oleic acid modification, exhibited poor dispersibility in the DVB monomer, leading to the coexistence of pure polymer particles (PPPs) alongside MPCPs containing encapsulated Fe_3_O_4_ (as seen in Figure 5h). In contrast, oleic acid-modified highly hydrophobic MNPs, possessing enhanced compatibility with the DVB monomer, well dispersed within the DVB, resulting in the formation of pure MPCPs with encapsulated Fe_3_O_4_ (Figure 5a–g). In addition, the content of SDS as an emulsifier and CA as a stabilizer played a critical role in determining the final morphology and encapsulation rate of the MPCPs. In this study, the SDS content was maintained below its critical micelle concentration (CMC) of 8 mM to minimize nucleation in the aqueous phase and micelle formation, which is crucial for achieving encapsulated MPCPs. However, excessively low SDS concentrations could compromise the stability of the mini-emulsion. As shown in Figure 5a, low SDS content (0.02 g) resulted in irregularly shaped particles, although without the presence of PPPs or BMPs. As the SDS content increased, the shape of MPCPs became more regular; the size became smaller and more uniform. When the SDS content was increased to 0.06 g, slightly below its CMC, a large number of regular spheres with diameters around 200 ± 25 nm were obtained, as shown in Figure 5c. A high-magnification TEM image of sample S-3 (Figure 5d) clearly reveals the encapsulation structure of the MPCPs. The image confirms that all Fe_3_O_4_ nanoparticles were effectively encapsulated within the polymer spheres, with no pure polymer particles (PPPs) or bare magnetic particles (BMPs) detected. Moreover, a poly(divinylbenzene) (PDVB) layer several nanometers thick was also observed on the outermost surface of the MPCPs (highlighted by an arrow in the image), further validating the successful encapsulation and offering the potential for surface modification.

These findings indicate that achieving stable mini-emulsions and producing MPCPs with more uniform sizes requires formulating the sodium dodecyl sulfate (SDS) concentration near its critical micelle concentration (CMC). Using this optimized approach, MPCPs can be successfully fabricated with Fe_3_O_4_ nanoparticles encapsulated inside, like sample S-3, with a diameter of approximately 200 ± 25 nm.

However, achieving smaller diameters and monodisperse particle size distributions remains a priority for expanding the application range of MPCPs, particularly in bio-related fields. This can be effectively addressed by optimizing the CA content. CA functions as a stabilizer in the mini-emulsion system, reducing the surface tension of the DVB monomer and facilitating the formation of smaller, more uniform monomer droplets. Following polymerization, these droplets transform into solid MPCPs, thus influencing the final particle size and uniformity. As demonstrated by comparing Figure 5c,e,g, increasing the CA content from 0.2 g to 0.3 g resulted in a reduction in the average MPCP size from approximately 200 nm to around 100 nm, accompanied by a narrower size distribution (see Table 2). Further increasing the CA content to 0.4 g yielded even more uniform particle sizes and enhanced encapsulation efficiency (Figure 5f,g).

### 3.3. Formation Mechanism of MPCPs

**Scheme 2:**
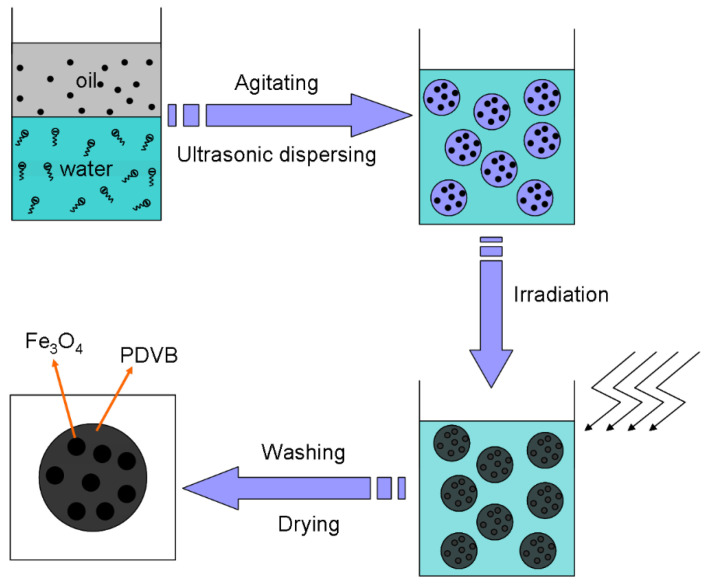
MPCP preparation procedure.

Based on the results, we propose a formation mechanism for MPCPs, illustrated in Scheme 2. Mini-emulsion polymerization proves to be an effective method for encapsulating MNPs within polymers. This is attributed to the inherent characteristics of the mini-emulsion system, which minimizes the presence of micelles in the aqueous phase and reduces the likelihood of nucleation in the aqueous phase, as described in other works utilizing this technique. Polymerization predominantly occurs within the small monomer droplets. Furthermore, the combined action of the emulsifier and stabilizer, coupled with ultrasonic treatment, promotes the formation of mini droplets of monomers containing Fe_3_O_4_. As a result, enhanced dispersibility of Fe_3_O_4_ within the monomer droplets leads to the formation of MPCPs encapsulating a high content of Fe_3_O_4_ following polymerization. Therefore, the surface modification of Fe_3_O_4_ is critical in this process, as highlighted throughout this study. Moreover, the fast polymerization rate and high degree of crosslinking of DVB are more beneficial to suppressing the phase separation between Fe_3_O_4_ and PDVB, which is also one of our purposes for using DVB [22]. Under sufficient agitating and moderate ultrasonic dispersing, we can obtain a stable mini-emulsion. Then, γ-ray irradiation was used to initiate the polymerization of the DVB monomer and solidify the structure formed during mini-emulsification. Upon γ-ray irradiation of the stabilized mini-emulsion, the polymerization process is initiated. The radiolysis of water leads to the generation of various active intermediates, including hydrated electrons (e-aq), hydrogen radicals (H-), and hydroxyl radicals (-OH). These reactive species then trigger the polymerization of the DVB monomer, resulting in the formation of PDVB nanospheres that encapsulate the Fe_3_O_4_ nanoparticles.

### 3.4. Properties Characterizations of MPCPs

**Figure 6:**
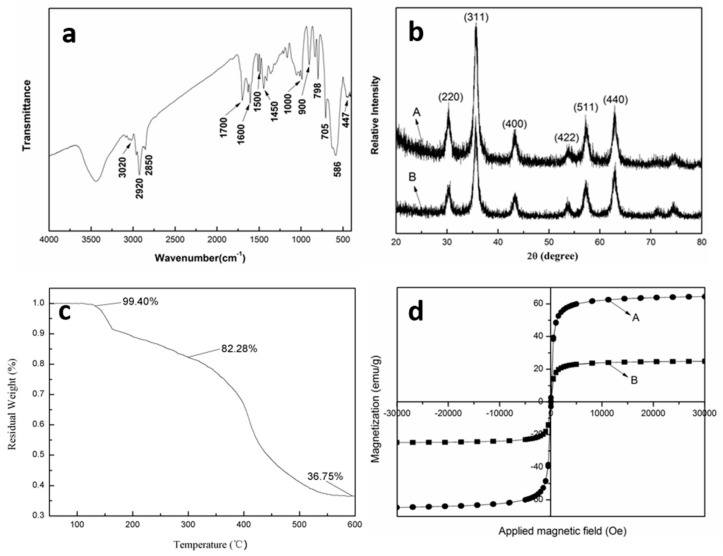
(**a**) FTIR spectrum S-5 in Table 1, (**b**) XRD of S-5 in Table 1 (A) and MNPs (B), (**c**) TGA curve S-5 in Table 1 and (**d**) Hysteresis loop measurement of MNPS, (A) and S-5 in Table 1 (B).

Moreover, a series of tests was performed on MPCPs, as shown in S-5 in Table 1, which is the best sample we obtained in this work. The results are shown in Figure 6.

The FTIR spectrum of S-5, shown in Figure 6a, confirms the presence of PDVB. The peaks at 705 and 798 cm^−1^ correspond to the bending vibrations of the C-H bond (δC-H) in the benzene ring. The bands at 1450, 1500, and 1600 cm^−1^ are attributed to the stretching vibrations of the C-C bond (νC-C) in the benzene ring. A peak at 3020 cm^−1^ is associated with the C-H stretching vibration (νC-H) of the benzene ring. The bands at 2850 and 2920 cm^−1^ correspond to the stretching vibrations of saturated C-H bonds (νC-H), likely from -CH_2_ groups in PDVB or -R groups in oleic acid. The band at 1700 cm^−1^ is attributed to the stretching vibration of the C=O group in oleic ester. The peaks at 1000 and 900 cm^−1^ indicate the presence of C-O-C stretching vibrations (νC-O-C) from oleic acid. The bands at 586 and 447 cm^−1^ confirm the presence of Fe^2^⁺–O^2−^ and Fe^3^⁺–O^2−^, which are characteristic of the chemical structure of MNPs.

To further identify the MNPs, XRD analysis was conducted on both MNPs and S-5 (Figure 6b). The diffraction peaks for MNPs (Figure 6b-B) align with the face-centered magnetite (Fe_3_O_4_) crystallite structure, consistent with the literature (JCPDS card file no. 74-0748). Identical peaks were observed for S-5, indicating that the crystallinity of MNPs remained unchanged throughout the preparation process. The baseline of the XRD for S-5 shows a slight increase in the low 2θ range, attributable to the presence of PDVB.

The TGA of S-5 is shown in Figure 6c. A weight loss of 17.72% up to 300 °C corresponds to the removal of water, surfactant, stabilizer, and absorbed oleic acid. The subsequent weight loss of 45.53% between 300 °C and 600 °C is attributed to the decomposition of PDVB. The residual weight of 36.75% represents the pure MNPs. Therefore, the MNP content in S-5 is calculated to be 44.66% (36.75/82.28 × 100%). This high magnetic content is beneficial for the application of these composite microspheres, especially in fields such as biology and pharmaceuticals [23].

Magnetic characterization of MNPs and walnut-like microspheres S-5 was performed at 300 K (Figure 6d). Both bare MNPs and S-5 microspheres exhibited zero remnant magnetization at 300 K, indicating superparamagnetic behavior. This property helps prevent aggregation of S-5 in the absence of an external magnetic field. The specific saturation magnetization of S-5 was approximately 20 emu/g, slightly lower than the typical range of 30–80 emu/g observed for magnetic nanoparticles in biomedical applications [24]. Further improvements could be made by increasing the MNP content.

## 4. Conclusions

In this work, we successfully prepared magnetic polymer particles encapsulating Fe_3_O_4_ via γ-ray irradiated mini-emulsion polymerization. Here, a mini-emulsion acting like a soft template can efficiently control prepared polymer particle size and size distribution. Synthesized Fe_3_O_4_ nanoparticles need to be further modified and changed from hydrophilic to hydrophobic; then, they can be dispersed well in monomer before polymerization, which was proven to be significant in forming a high degree of encapsulation. PPPs and BMPs can be completely avoided by selecting preparation conditions. The results show that SDS concentration must be close to its CMC in water in order to obtain a stable mini-emulsion and narrow the droplet size distribution. CA content played an essential role in controlling the size and distribution of MPCPs. In our work, higher SDS and CA content resulted in MPCPs with smaller diameters and narrower size distribution. TEM and FESEM analyses confirmed the successful formation of MPCPs with Fe_3_O_4_ encapsulated within the polymer matrix. Magnetic measurements demonstrated that the resulting MPCPs exhibit superparamagnetic behavior and strong magnetic induction at room temperature, properties that are highly advantageous for various bio-applications.

## Figures and Tables

**Table 1:** Experimental conditions for fabricating MPCPs.

MPCPs	H_2_O (mL)	SDS (g)	DVB (g)	CA (g)	MNPs (g) (Modified)	MNPs (g) (Unmodified)
S-1	30	0.02	3	0.20	0.10	/
S-2	30	0.04	3	0.20	0.10	/
S-3	30	0.06	3	0.20	0.10	/
S-4	30	0.06	3	0.30	0.10	/
S-5	30	0.06	3	0.40	0.10	/
S-6	30	0.06	3	0.20	/	0.10
S-7	30	0.06	3	0.2	/	/

**Table 2:** Size for different samples listed in Table 1.

MPCPs	Average Size (nm)	Standard Deviation (nm)
S-1	500	150
S-2	400	100
S-3	200	25
S-4	100	15
S-5	80	10
S-6	200	150
S-7	50	5

## Data Availability

The original contributions presented in this study are included in the article.

## References

[1] Dong X., Zheng Y., Huang Y., Chen X., Jing X. (2010). Synthesis and characterization of multifunctional poly (glycidyl methacrylate) microspheres and their use in cell separation. Anal. Biochem..

[2] Hong X., Chu X., Zou P., Liu Y., Yang G. (2010). Magnetic-field-assisted rapid ultrasensitive immunoassays using Fe_3_O_4_/ZnO/Au nanorices as Raman probes. Biosens. Bioelectron..

[3] Sur K., McFall S.M., Yeh E.T., Jangam S.R., Hayden M.A., Stroupe S.D., Kelso D.M. (2010). Immiscible phase nucleic acid purification eliminates PCR inhibitors with a single pass of paramagnetic particles through a hydrophobic liquid. J. Mol. Diagn..

[4] Chen F., Shi R., Xue Y., Chen L., Wan Q.H. (2010). Templated synthesis of monodisperse mesoporous maghemite/silica microspheres for magnetic separation of genomic DNA. J. Magn..

[5] Sureshkumar M., Lee C.K. (2011). Polydopamine coated magnetic-chitin (MCT) particles as a new matrix for enzyme immobilization. Carbohydr. Polym..

[6] Brugger E.C., Prayer D. (2011). Development of gastroschisis as seen by magnetic resonance imaging. Ultrasound Obstet. Gynecol..

[7] Maity D., Chandrasekharan P., Yang C.T., Chuang K.H., Shuter B., Xue J.M., Ding J., Feng S.S. (2010). Facile synthesis of water-stable magnetite nanoparticles for clinical MRI and magnetic hyperthermia applications. Nanomedicine.

[8] Gorti V.M., Shang H., Wereley S.T., Lee G.U. (2008). Immunoassays in nanoliter volume reactors using fluorescent particle diffusometry. Langmuir.

[9] Guchhait A., Rath A.K., Pal A.J. (2009). Hybrid core−shell nanoparticles: Photoinduced electron-transfer for charge separation and solar cell applications. Chem. Mater..

[10] Liao Z., Wang H., Lv R., Zhao P., Sun X., Wang S., Su W., Niu R., Chang J. (2011). Polymeric liposomes-coated superparamagnetic iron oxide nanoparticles as contrast agent for targeted magnetic resonance imaging of cancer cells. Langmuir.

[11] Yanase N., Noguchi H., Asakura H., Suzuta T. (1993). Preparation of magnetic latex particles by emulsion polymerization of styrene in the presence of a ferrofluid. J. Appl. Polym. Sci..

[12] Wormuth K. (2001). Superparamagnetic latex via inverse emulsion polymerization. J. Colloid Interface Sci..

[13] Deng Y.H., Yang W.L., Wang C.C., Fu S.K. (2003). A novel approach for preparation of thermoresponsive polymer magnetic microspheres with core–shell structure. Adv. Mater..

[14] Wang P.C., Chiu W.Y., Lee C.F., Young T.H. (2004). Synthesis of iron oxide/poly (methyl methacrylate) composite latex particles: Nucleation mechanism and morphology. J. Polym. Sci. Part A Polym. Chem..

[15] Csetneki I., Faix M.K., Szilagyi A., Kovacs A.L., Nemeth Z., Zrinyi M. (2004). Preparation of magnetic polystyrene latex via the miniemulsion polymerization technique. J. Polym. Sci. Part A Polym. Chem..

[16] Zaitsev V.S., Filimonov D.S., Presnyakov I.A., Gambino R.J., Chu B. (1999). Physical and chemical properties of magnetite and magnetite-polymer nanoparticles and their colloidal dispersions. J. Colloid Interface Sci..

[17] Vestal C.R., Zhang Z.J. (2002). Atom transfer radical polymerization synthesis and magnetic characterization of MnFe2O4/polystyrene core/shell nanoparticles. J. Am. Chem. Soc..

[18] Deng Y., Wang L., Yang W., Fu S., Elaıssari A. (2003). Preparation of magnetic polymeric particles via inverse microemulsion polymerization process. J. Magn. Magn. Mater..

[19] Xu Z.Z., Wang C.C., Yang W.L., Deng Y.H., Fu S.K. (2004). Encapsulation of nanosized magnetic iron oxide by polyacrylamide via inverse miniemulsion polymerization. J. Magn. Magn. Mater..

[20] Landfester K., Ramirez L.P. (2003). Encapsulated magnetite particles for biomedical application. J. Phys. Condens. Matter.

[21] Yang S., Liu H., Zhang Z. (2008). Fabrication of novel multihollow superparamagnetic magnetite/polystyrene nanocomposite microspheres via water-in-oil-in-water double emulsions. Langmuir.

[22] de Oliveira Reis M., de Sousa R.G., Batista A.D.S.M. (2022). Synthesis and characterization of poly (styrene-co-divinylbenzene) and nanomagnetite structures. MethodsX.

[23] Lu A.H., Salabas E.E., Schüth F. (2007). Magnetic nanoparticles: Synthesis, protection, functionalization, and application. Angew. Chem. Int. Ed..

[24] Sun C., Lee J.S., Zhang M. (2008). Magnetic nanoparticles in MR imaging and drug delivery. Adv. Drug Deliv. Rev..

